# A New Method for Predicting the Liquid Dynamic Viscosity of Biodiesel-Related Esters Based on the Corresponding State Principle

**DOI:** 10.1021/acsomega.5c03190

**Published:** 2025-07-17

**Authors:** Maxwell Risseli Laurentino da Silva, Alanderson Arthu Araújo Alves, Nathan Sombra Evangelista, Rafael Leandro Fernandes Melo, Frederico Ribeiro do Carmo, Osvaldo Chiavone-Filho

**Affiliations:** † Departamento de Engenharia Química, NUPEG, 28123 Universidade Federal do Rio Grande do Norte, Campus Universitário, Lagoa Nova, 59078-970 Natal, Rio Grande do Norte, Brazil; ‡ Núcleo de Pesquisa em Economia de Baixo Carbono, Departamento de Engenharia e Tecnologia, Centro de Engenharia, Universidade Federal Rural do Semi-Árido, Campus Leste, 59625-900 Mossoró, Rio Grande do Norte, Brazil; § Grupo de Pesquisa em Termofluidodinâmica Aplicada, Departamento de Engenharia Química, Centro de Tecnologia, Universidade Federal do Ceará, Campus do Pici, Bloco 709, 60455-760 Fortaleza, Ceará, Brazil; ∥ Departamento de Engenharia Química e de Materiais, Universidade Federal de Lavras, Campus Universitário, 37200-000 Lavras, Minas Gerais, Brazil

## Abstract

This work proposes a new model based on the three-parameter corresponding states principle (CSP) for estimating the dynamic viscosity of biodiesel-related esters in the liquid state. A Tait-like equation was employed to extend the model to high-pressure conditions. The model’s parameters were fitted to 249 experimental viscosity data points from 11 biodiesel-related esters, with 204 obtained under high-pressure conditions. The average absolute relative deviations were 7.94% for high-pressure conditions and 12.95% for atmospheric conditions. The proposed method was rigorously compared with 13 of the most consolidated models available in the literature for the same property (9 based on the group contribution concept, 3 based on the CSP, and 1 based on both methodologies). Considering all criteria of accuracy and physical consistency, the proposed model is recommended for future applications.

## Graphical abstract

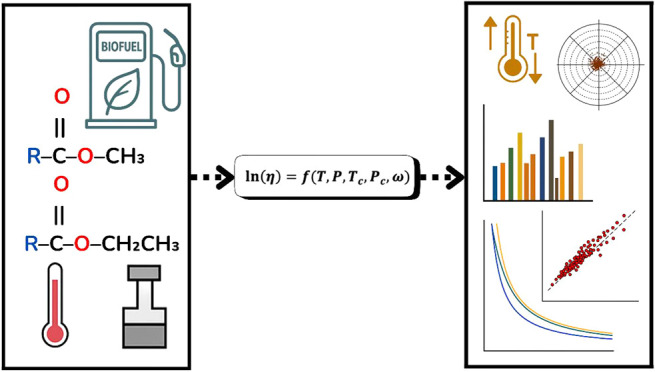

## 1 Introduction

The world still has significant dependence on fossil fuels and their derivatives. According to International Energy Agency data,^1^ until 2022, fossil fuels and their derivatives accounted for approximately 75% of all primary sources used for energy supply. For instance, 60% of the economy is related to the transportation sector. Diesel engines are the major contributors to this consumption and are responsible for high carbon dioxide emissions and other environmentally harmful gases.^2^

Biodiesel is a mixture of long-chain fatty acid alkyl esters (FAAEs) that is industrially obtained by a transesterification reaction. It is an alternative to diesel and can be used in diesel engines without further modifications. Biodiesel also presents many advantages, such as low flammability, less particulate matter in its combustion products, free of sulfur and aromatics, and a high cetane number.^3–5^

The knowledge of biodiesel thermophysical properties such as viscosity is fundamental for the development and optimization process and a better understanding of variables that can affect the efficiency of the combustion phenomenon.^6^ Furthermore, biodiesel properties are also important in the development, optimization, and control of processes in biorefineries.^7^

Experimental investigations into the effects variables such as viscosity have on biodiesel emissions have been exhaustively carried out. However, performing such analyses for biodiesel derived from different feedstocks becomes impractical due to the wide variety of raw materials. Therefore, computational fluid dynamics (CFD) has emerged as an effective approach for carrying out these analyses.^2^ In CFD studies, the use of accurate predictive models for viscosity is essential to ensure reliable simulation results.^8^ In the literature, researchers frequently evaluate predictive models for the liquid viscosity of fatty acid methyl esters (FAMEs) and fatty acid ethyl esters (FAEEs), and subsequently extend their applicability to biodiesel via appropriate mixing rules. Liquid viscosity, which quantifies a fluid’s resistance to flow,^9^ significantly influences the performance of diesel engines. Elevated viscosity levels can hinder fuel atomization, increase injection pump pressures, and ultimately compromise the combustion efficiency. These effects often lead to higher pollutant emissions due to incomplete combustion reactions.^2,10,11^

Even though they are essential for the liquid phase, experimental viscosity data in a wide range of temperatures and pressures are scarce for FAAEs, and their measurements are expensive and time-consuming. Because of that, predictive models are a simple alternative to overcome the issues related to experimental investigations. Moreover, predictive models can be applied in CFD simulations to produce reliable results.^6,8^

The most straightforward and rapid way to estimate liquid viscosity is by using Group Contributions (GC) and the Corresponding State Principle (CSP) models.^12^ The GC concept establishes that the macroscopic properties of the substances can be calculated by summation of individual groups that constitute the molecule, also called functional groups. This class of models is a helpful tool because the primary information needed is the structure of the species of interest. Another simple methodology to estimate properties is by using models from CSP, which states that substances at the same reduced variables (e.g., reduced temperature, pressure, and volume) must present similar behavior.^13^

In this work, we propose a new model based on CSP to predict the dynamic liquid viscosity (η) of FAMEs and FAEEs. Thus, it minimizes the number of fitted parameters, enhances simplicity, and offers satisfactory outcomes and accuracy. To develop the model, we created an extensive database containing experimental viscosities of FAMEs and FAEEs in a wide range of temperatures and pressures. Additionally, we compared its output results with the other 13 predictive models based on GC and CSP concepts. Finally, an extension to high-pressure conditions was proposed using a Tait-like equation with four fitted parameters.

## 2 Methodology

### 2.1 FAAEs Experimental Database

Accurate experimental data on physical properties is fundamental to the model’s development. Experimental data are used as comparison criteria to evaluate existing models and to develop new ones.^14,15^ Because of that, an experimental database containing experimental values of viscosities of fatty acid methyl esters and fatty acid ethyl esters that may occur in biodiesel has been created. Table 1 summarizes all collected data, and further details are available in the Supporting Information.

**Table 1:** Summary of the Experimental Dynamic Viscosity Data of FAMEs and FAEEs

compound	^a^acronym	Tmin/K	Tmax/K	Pmin/MPa	Pmax/MPa	number of data (accepted)	number of data (rejected)	references
methyl butyrate	ME-C4:0	273.10	373.07	0.10	0.10	37	2	^16–19^
methyl valerate	ME-C5:0	283.15	343.12	0.10	0.10	14	0	^19–22^
methyl caproate	ME-C6:0	283.10	372.02	0.10	30.01	60	6	^20,23–25^
methyl enanthate	ME-C7:0	283.10	361.47	0.10	30.03	63	16	^20–22,26^
methyl caprylate	ME-C8:0	263.05	372.02	0.10	30.06	77	19	^20,24–26^
methyl pelargonate	ME-C9:0	293.14	360.96	0.10	30.03	42	18	^20,27,28^
methyl caprate	ME-C10:0	263.05	372.02	0.10	200.00	106	58	^20,23–25^
methyl undecanoate	ME-C11:0	293.14	343.12	0.10	0.10	3	0	^20^
methyl laurate	ME-C12:0	278.10	372.02	0.10	30.29	109	9	^20,23,25,26^
methyl tridecanoate	ME-C13:0	293.14	343.12	0.10	0.10	3	0	^20^
methyl myristate	ME-C14:0	293.14	372.02	0.10	100.00	80	12	^20,23,25,29^
methyl pentadecanoate	ME-C15:0	293.14	343.12	0.10	0.10	14	0	^34,44^
methyl palmitate	ME-C16:0	303.15	372.02	0.10	0.10	30	1	^20,25,27,31^
methyl palmitoleate	ME-C16:1	263.05	363.15	0.10	0.10	18	12	^27,32,33^
methyl heptanoate	ME-C17:0	310.93	333.12	0.10	0.10	2	0	^20^
methyl stearate	ME-C18:0	310.93	573.18	0.10	0.10	25	1	^20,23,25,31^
methyl oleate	ME-C18:1	263.05	353.15	0.10	0.10	51	2	^20,27,31,32^
methyl linoleate	ME-C18:2	263.05	353.15	0.10	0.10	35	3	^20,27,31,32^
methyl linolenate	ME-C18:3	263.05	373.15	0.10	0.10	32	10	^20,27,32,34^
methyl nonadecanoate	ME-C19:0	313.13	343.12	0.10	0.10	2	0	^20^
methyl arachidate	ME-C20:0	323.15	373.15	0.10	0.10	11	0	^33^
methyl gadoleate	ME-C20:1	278.15	373.15	0.10	0.10	20	0	^33^
methyl behenate	ME-C22:0	333.15	373.15	0.10	0.10	9	0	^33^
methyl erucate	ME-C22:1	278.15	363.15	0.10	0.10	18	0	^33^
methyl lignocerate	ME-C24:0	338.15	373.15	0.10	0.10	8	0	^33^
ethyl butyrate	EE-C4:0	288.15	348.12	0.10	0.10	32	2	^18,19,23,35^
ethyl valerate	EE-C5:0	293.15	313.15	0.10	0.10	4	0	^19,36^
ethyl caproate	EE-C6:0	288.15	358.15	0.10	0.10	14	14	^19,23,37,38^
ethyl enanthate	EE-C7:0	312.72	353.04	0.10	15.17	30	0	^39^
ethyl caprylate	EE-C8:0	263.05	358.15	0.10	15.24	52	0	^23,40–42^
ethyl caprate	EE-C10:0	263.05	353.15	0.10	200.00	84	50	^43^
ethyl laurate	EE-C12:0	273.10	353.65	0.10	15.20	71	3	^23,27,31,32^
ethyl myristate	EE-C14:0	283.15	353.15	0.10	100.00	74	8	^29^
ethyl palmitate	EE-C16:0	298.14	573.18	0.10	0.10	24	0	^23,27,31,44^
ethyl palmitoleate	EE-C16:1	293.15	310.14	0.10	0.10	3	0	^45^
ethyl stearate	EE-C18:0	310.14	573.18	0.10	0.10	20	1	^23,27,31,44^
ethyl oleate	EE-C18:1	263.05	363.15	0.10	0.10	51	0	^27,30–32^
ethyl linoleate	EE-C18:2	263.05	363.15	0.10	0.10	32	1	^27,32,33,45^
ethyl linolenate	EE-C18:3	263.05	373.15	0.10	0.10	35	0	^27,32,33,45^
ethyl arachidate	EE-C20:0	318.15	373.15	0.10	0.10	12	0	^33^
overall						1655	248	

a ME and EE denote methyl ester and ethyl ester, respectively; In C*x/y*, *x* and *y* denote the number of carbons and double bonds in the fatty acid chain, respectively.

A total of 1655 experimental viscosity values were collected for 25 FAMEs and 15 FAEEs, distributed in 47 references published between 1897 and 2020. The minimum and maximum temperatures and pressures covered were 263.0–573.18 K and 0.10–200.00 MPa. Most of the data are under atmospheric pressure (0.10 MPa), totaling 998 data, and the remaining 658 data are under this condition. Viscosity data under high pressure for ME-C18:1 and ME-C18:2 ^46^ were not included because these were obtained using an extrapolative methodology.

As it is broadly known and based on previous experiences^8,47^ when dealing with different data sets, multiple values may be provided for FAEEs from various references. Because of this, it is necessary to verify the agreement within these references because some variables, such as experimental apparatus/methodology and sample purities, can affect the uncertainty associated with the final value of η. Therefore, it was adopted a refinement procedure to select the best data for each FAME and FAEE in the database, and mitigate possible problems that could be associated with higher uncertainties.^48^

The current database contains η under diversified pressure conditions. Two different approaches were adopted to refine these data. At atmospheric conditions, the correlation proposed by Vogel^12^ expressed by eq 1, which only considers η as temperature-dependent, and the refinement must be carried out at constant pressure. Additionally, all data at pressures less than 0.13 MPa were considered under atmospheric conditions.

1 $$\ln\eta \left(T\right)=A+\frac{B}{T+C}$$

where A, B, and C are constants obtained by optimization, and *T* is the observed temperature in Kelvin.

For viscosity at high pressures, a Tait-like equation^49^ was used as presented in eqs 2 and 3.

2 $$\ln\eta \left(T,P\right)=\eta_{0}\left(T,P_{0}\right)\;e\mathrm{xp}\left[D\ln\left(\frac{E\left(T\right)+P}{E\left(T\right)+P_{0}}\right)\right]$$

3 $$E\left(T\right)=D_{0}+D_{1}T+D_{2}T^{2}$$

where *P*_0_ is the reference pressure (in this work, 0.10 MPa was selected), η_0_ is the viscosity calculated at *P*_0_, *P* denotes the observed pressure in MPa, and *D*_0_, *D*_1_, and *D*_2_ are also constants obtained by optimization. As seen in eq 2, when *P* = *P*_0_, the models return the viscosity at the reference pressure η_0_.

To perform both refinements, choosing the tolerance that should be followed as the refinement criteria was necessary. Although η is a property that does not require greater exactness than other thermodynamic properties, there are no reference values for it, only a few reports about accuracy from well-established methods.^50^

Therefore, considering the maximum uncertainty found in the experimental database, a value of 4.0% was chosen as a refinement criterion. Only refined FAEEs had, at minimum, the number of data higher than double the optimizable parameters^50^ (in eqs 1 or 2). The refinement results are presented in Table 1. Supporting Information provides further details, such as optimized parameters and the number of accepted and rejected data.

### 2.2 Predictive Models

As highlighted in Section 1, our goal was to compare the model proposed in this study with established models already described in the literature by other authors. These models are summarized in Table 2. The input parameters for the models in Table 2, including the functional group assignments for each GC model, are provided in the Supporting Information.

**Table 2:** Selected Models to Predict the Liquid Dynamic Viscosity

		^a^	^b^required inputs								
method	acronym	conceptual basis^a^	molecular structure	ρ	*M*_W_	*T*_*m*_	*T*_*b*_	*T*_*c*_	*P*_*c*_	*V*_*c*_	ω
joback and reid^51^	JR	GC	×		×						
orrick and erbar^13^	OR	GC	×	×	×						
thomas^52^	thomas	GC	×	×				×			
morris^12^	morris	GC	×					×			
hsu, sheu, and tu^53^	HST	GC	×						×		
yinghua et al.^54^	YPP	GC/CSP	×		×		×				
ceriani et al.^55^	CGC	GC	×		×						
souders^56^	souders	GC	×	×	×						
nannoolal et al.^57^	NRR	GC	×				×				
sastri and rao^58^	SR	GC	×				×	××			
van velzen et al.^59^	VCL	GC	×								
przezdziecki and sridhar^60^	PS	CSP		×		×		×	×	×	×
letsou and stiel^61^	LS	CSP			×			×			×

a GC: Group contribution; CSP: Corresponding States Principle.

b ρ: density of the liquid at specific temperature; *M*_W_: molecular weight; *T*_*m*_: melting temperature; *T*_*c*_: critical temperature; *P*_*c*_: critical pressure; *V*_*c*_: critical volume; ω: acentric factor.

The required parameters *T*_*b*_, *T*_*c*_, *P*_*c*_, and ω, used in this work, were taken from the database suggested by Evangelista et al.^62^ Melting temperatures used were taken from do Carmo et al.^6^ The Alvarez e Valderrama^63^ method was used for *V*_*c*_ estimations. Additionally, for those models that require ρ as input, the CSP model proposed by Yen and Woods^64^ was used due to its performance for systems composed of polar compounds.

### 2.3 The New CSP Model

The three-parameter Corresponding States Principle (CSP) states that if two fluids have identical acentric factors (ω), at the same reduced temperature (*T*_*r*_) and reduced pressure (*P*_*r*_), their equilibrium properties will also be identical.^65–67^ Thus, any reduced property of a fluid can be expressed as a function of three parameters: *T*_*r*_, *P*_*r*_, and ω. Teja and Rice^68^ derived the following expression for viscosity:

4 $$\ln\left(\xi \eta \right)=\ln{\left(\xi \eta \right)}^{R_{1}}+\frac{\omega -\omega^{R_{1}}}{\omega^{R_{2}}-\omega^{R_{1}}}\left[\ln{\left(\xi \eta \right)}^{R_{2}}-\ln{\left(\xi \eta \right)}^{R_{1}}\right]$$

where *R*_1_ and *R*_2_ denote the properties of reference fluids 1 and 2, respectively. Viscosities of reference fluids (η^*R*_*j*_^) are calculated by using eq 5.

5 $$\ln{\left(\xi \eta \right)}^{R_{j}}=A+\frac{B}{T_{r}},j=\left[1,2\right]$$

where A and B are constants obtained from fitting experimental data and *T_r_* is the reduced temperature defined as *T*/*T*_*c*_. The term ξ is equivalent to the critical viscosity. Teja et al.^69^ suggested the following equation to calculate it

6 $$\xi =\frac{T_{c}^{1/6}}{M_{W}^{1/2}P_{c}^{2/3}}$$

A computational procedure was developed to determine the optimal values of parameters A and B in eq 5 for each of the 40 individual FAAEs with available experimental data in our database. Subsequently, all possible pairs among these esters were evaluated as reference fluids using eq 4. Based on this evaluation, ME-C4:0 and ME-C24:0 were selected as reference fluids 1 and 2, respectively. The resulting eq 5 expressions for these two reference esters are shown below

7 $$\ln{\left(\xi \eta \right)}^{R_{1}}=-22.49+\frac{1.99}{T_{r}}$$

8 $$\ln{\left(\xi \eta \right)}^{R_{2}}=-22.99+\frac{2.76}{T_{r}}$$

Eqs 7 and ([Disp-formula eq8]) provide η values in mPa·s. We will refer to the proposal as “da Silva 2f” hereafter.

The parameters were obtained by minimizing the following objective function

9 $$\mathrm{OF}=\sum_{i}{\left(\frac{\eta_{i}^{\exp}-\eta_{i}^{\mathrm{calc}}}{\eta_{i}^{\exp}}\right)}^{2}$$

where η_*i*_^exp^ and η_*i*_^calc^ represent the experimental and calculated dynamic viscosity, respectively. Eq 9 summation includes all available experimental data points for each FAAE. Optimization was conducted using a modified Levenberg–Marquardt algorithm for nonlinear least-squares regression, implemented through a Python script utilizing the *least_squares* routine from the SciPy library.^70^ The optimization process was repeated with different initial estimates for parameters A and B to guarantee convergence to a global minimum.

In certain applications, accurate dynamic viscosity values of FAAEs under high-pressure conditions are necessary. To accommodate such cases, the proposed model was extended to elevated pressures by calculating the term η_0_ in eq 2 using eqs 4–([Disp-formula eq8]). The parameters *D*_0_, *D*_1_, *D*_2_, and *C* were treated as global constants obtained through fitting to experimental data from selected compounds.

## 3 Evaluation Procedure

### 3.1 Statistical Performance Indicators

To evaluate the proposed model and compare it with the other models presented in Table 2, the statistical indicator average absolute relative deviation (%AARD) was used

10 $$\%\mathrm{AARD}=\frac{100}{N_{\mathrm{data}}}\sum_{i}^{N_{\mathrm{data}}}\left|\frac{\eta_{i}^{\mathrm{calc}}-\eta_{i}^{\exp}}{\eta_{i}^{\exp}}\right|$$

The superscripts calc and exp denote dynamic viscosity calculated by the models and experimental, respectively. *N*_data_ is the number of experimental data. The %AARD was chosen because it can provide accuracy and quantify the degree of data scatter of the models.^71^

### 3.2 Physical Behavior Evaluation

As noted in our earlier studies,^6,8,15,62^ a predictive model must demonstrate accuracy and align with empirical observations, exhibiting physical consistency. Hence, this section outlines two methodologies designed to assess the physical consistency of all predictive models.

For an FAAE of the same class (FAMEs or FAEEs), with an equal amount of unsaturation in the chain, it is observed that η increases as the number of carbons increases. The homologous series test aims to evaluate the performance of all models submitted to eq 11 through 34 homologous pairs of FAAEs analysis that may occur in biodiesel. Further information is provided in the Supporting Information.

11 $$\eta_{\left(i+1\right)}>\eta_{\left(i\right)}\mathrm{at}\;\mathrm{the}\;\mathrm{same}\;\mathrm{temperature}$$

The terms (*i*+1) and (*i*) denote adjacent members of a homologous series. This test was performed between the *T*_*m*_ of the member (*i*+1) and *T*_*b*_ of the member (*i*). This temperature interval was selected to fairly evaluate GC models, as most of these models have been originally proposed for application within this specific range.

As criteria, if the ratio (η_(*i*)_ – η_(*i*+1)_)/η_(*i*+1)_ calculated by each model was greater than the more significant uncertainty found in the experimental database, which was 4.0%, at least one temperature, the model was considered inconsistent and will not pass to the next test.

The most accurate model for FAMEs is not necessarily the best for FAEEs. For this reason, “*n* × *n*” packages were created, where *n* corresponds to the number of models passed in the homologous series test. Each package analyzes one possible combination between the models, wherein one package (*i*–*j*) represents one model for FAMEs (*i*) and another for FAEEs (*j*). The accuracy of all packages was calculated using eq 12

12 $$\%\mathrm{ARRD}\left(i-j\right)=\frac{100}{N_{\mathrm{data}}}\left(\sum_{k=1}^{N_{\mathrm{FAME}}}\left|\frac{\eta_{k}^{\mathrm{calc}}-\eta_{k}^{\exp}}{\eta_{k}^{\exp}}\right|+\sum_{k=1}^{N_{\mathrm{FAEE}}}\left|\frac{\eta_{k}^{\mathrm{calc}}-\eta_{k}^{\exp}}{\eta_{k}^{\exp}}\right|\right)$$

The summation in eq 12 for models *i* and *j* covers the amount of data for FAMEs (*N*_FAME_) and FAEEs (*N*_FAEE_).

Similar to the homologous series, experimental observations show that when comparing members with the same chain length and unsaturation number and from different classes (FAMEs/FAEEs), the η value for an EE member is always higher than that for an ME member. Hence, all packages were submitted to eq 13. The procedure was performed between *T*_*m*_ of EE member and *T*_*b*_ of ME members for 34 ME/EE pairs of FAAEs. In addition, the ratio (η_ME_ – η_EE_)*/η*_EE_ calculated by each model was greater than 4.0%, at least one temperature, the package was considered inconsistent.

13 $$\eta_{\left(\mathrm{EE}\right)}>\eta_{\left(\mathrm{ME}\right)}\mathrm{at}\;\mathrm{the}\;\mathrm{same}\;\mathrm{temperature}$$

Initially, all predictive models were submitted to statistical and physical consistency analysis for only atmospheric conditions. After identifying the most accurate and physically consistent models, these were applied to predict the η values for FAAEs at elevated pressures.

## 4 Results and Discussion

### 4.1 Evaluating Optimum Models for FAAEs at Atmospheric Conditions

The proposed model (da Silva 2f) and the other 13 models mentioned in Section 2.3 were tested at atmospheric conditions (*P* ≤ 0.13 MPa) for all FAAEs in the experimental database. The performance of the models studied is presented in Table 3. With some exceptions, the GC models showed better predictions than CSP. This result is not surprising, considering that this class of models is more suggested at the temperature range: *T*_*m*_ *≤T ≤ T*_*b*_. Further details can be found in the Supporting Information.

**Table 3:** %AARD for Liquid Viscosity Obtained Using GC and CSP Models

	models													
FAAE	JR (%)	OR (%)	thomas (%)	Morris (%)	HST (%)	YPP (%)	CGC (%)	souders (%)	NRR (%)	SR (%)	VCL (%)	PS (%)	LS (%)	da Silva 2f (%)
ME-C4:0	5.93	4.27	3.67	3.00	1.93	5.39	6.31	74.16	9.08	1.94	1.08	114.04	23.37	1.04
ME-C5:0	11.98	1.95	1.69	12.57	4.88	3.55	1.17	47.12	14.89	5.66	1.85	109.16	34.40	4.06
ME-C6:0	10.57	2.82	7.87	25.66	6.41	3.56	1.37	96.66	12.18	4.31	5.22	108.52	35.84	8.50
ME-C7:0	11.35	3.38	5.53	25.57	6.49	3.80	1.49	148.47	12.29	10.09	6.63	106.79	45.98	0.92
ME-C8:0	7.79	3.16	5.55	27.92	10.03	4.93	2.75	133.35	8.41	14.55	10.78	105.85	50.24	4.44
ME-C9:0	9.18	2.02	12.08	37.18	14.23	5.31	1.76	193.81	10.66	27.32	10.34	105.24	53.61	6.22
ME-C10:0	8.61	3.28	9.22	36.93	16.86	8.07	1.51	171.55	4.11	22.53	11.56	103.75	63.51	13.51
ME-C11:0	9.82	3.24	18.16	50.76	20.02	5.83	0.59	175.46	8.90	54.57	10.77	103.22	66.62	12.65
ME-C12:0	9.45	3.19	23.33	59.78	29.32	7.01	1.87	114.86	4.10	44.13	11.46	102.68	72.04	18.14
ME-C13:0	10.84	4.22	32.34	71.81	23.75	6.24	1.02	139.71	5.67	78.93	10.73	102.27	73.29	13.88
ME-C14:0	10.40	4.37	32.91	71.30	34.15	5.19	2.11	70.89	3.89	123.32	11.34	102.11	75.98	21.93
ME-C15:0	21.54	13.67	51.48	96.07	28.62	8.35	1.42	76.35	8.81	182.44	3.41	101.70	77.87	16.70
ME-C16:0	15.86	8.87	56.19	102.66	27.58	7.09	1.83	95.03	7.53	138.55	7.82	101.83	77.21	12.65
ME-C16:1	13.72	7.98	64.88	109.33	36.71	6.29	2.60	62.51	4.66	154.20	9.23	101.97	73.58	15.08
ME-C17:0	24.15	12.14	4849.08	109.68	13183.29	30.71	13.74	88.50	24.48	243.93	15.86	101.53	78.49	18.50
ME-C18:0	32.27	24.49	105.46	160.75	25.40	22.17	12.72	80.92	19.10	251.29	5.98	102.78	72.12	8.92
ME-C18:1	15.74	13.15	85.00	126.41	40.04	13.48	2.60	45.81	5.64	252.95	14.23	101.08	82.55	11.15
ME-C18:2	24.87	7.75	6004.54	116.82	12518.62	18.56	4.41	65.79	11.53	314.66	11.92	101.19	80.24	7.09
ME-C18:3	33.68	3.54	283064.11	96.72	2255881.32	35.52	4.15	73.77	22.99	338.85	40.70	101.69	74.14	24.86
ME-C19:0	27.12	12.98	11501433.88	54.48	340107486.21	47.19	6.07	47.65	25.00	396.39	54.65	101.18	82.68	35.69
ME-C20:0	19.70	13.23	117.28	166.32	39.73	13.71	0.72	50.01	4.55	303.15	7.16	101.53	78.30	10.65
ME-C20:1	13.35	8.67	126.81	166.89	43.59	17.02	0.40	51.50	3.00	316.44	22276.55	101.20	81.57	7.52
ME-C22:0	21.61	9.11	7381.37	161.44	9348.87	31.61	1.88	44.89	12.70	459.28	26552.65	101.35	79.93	7.10
ME-C22:1	15.18	10.26	174.89	207.44	49.48	29.08	0.83	85.53	5.63	549.33	26837.00	100.89	84.45	3.93
ME-C24:0	37.12	21.19	10535.81	209.68	10454.05	42.91	3.94	27.51	15.30	743.73	34231.10	101.16	81.89	13.45
EE-C4:0	18.23	14.46	240.21	258.13	55.23	47.49	1.31	189.94	10.63	1226.09	32715.06	110.81	30.52	0.29
EE-C5:0	15.82	4.68	1.91	9.79	8.22	3.57	2.26	80.40	2.90	4.33	3.02	107.81	39.78	1.78
EE-C6:0	21.76	9.37	3.28	20.60	14.91	2.73	4.21	72.33	1.81	5.46	4.11	109.02	29.62	3.04
EE-C7:0	43.39	30.05	26.12	48.76	20.93	24.19	24.95	143.84	24.90	42.42	20.86	107.62	37.08	19.63
EE-C8:0	19.57	7.60	9.48	29.71	2.57	9.39	6.29	78.31	5.01	31.94	2.51	104.80	52.68	1.85
EE-C10:0	20.50	10.44	13.24	40.24	1.88	6.44	6.57	127.83	5.87	29.58	2.30	103.40	63.76	2.29
EE-C12:0	19.54	11.46	21.80	54.41	13.64	4.82	3.93	179.98	5.88	51.69	4.33	102.18	75.27	9.66
EE-C14:0	20.16	11.77	36.25	78.47	29.31	5.62	2.19	95.41	4.48	59.28	3.47	101.76	77.76	17.60
EE-C16:0	21.86	17.64	71.06	112.80	33.28	13.26	1.64	51.90	9.30	234.46	8.95	103.09	70.63	11.83
EE-C16:1	35.31	13.74	5858.13	124.72	11641.67	25.70	2.22	64.47	23.55	303.02	22.36	101.11	83.37	3.98
EE-C18:0	24.20	20.59	96.39	135.32	40.81	21.18	1.49	43.31	11.45	418.37	9.15	102.93	71.47	11.28
EE-C18:1	38.24	17.93	8322.63	160.52	10303.64	34.30	5.69	50.57	24.69	483.01	20.82	101.03	84.43	7.88
EE-C18:2	40.51	8.05	417820.35	130.66	1731664.19	55.08	4.59	50.32	36.01	533.51	45.19	101.24	81.35	26.80
EE-C18:3	42.32	3.78	23942321.02	96.20	296516627.72	79.00	4.98	51.57	46.28	588.80	72.44	101.56	76.73	53.63
EE-C20:0	23.52	17.15	155.82	194.68	44.54	34.98	1.34	72.44	14.20	774.22	26056.56	101.40	79.81	5.50
FAMEs	15.31	6.29	596376.10	75.55	17265600.26	14.56	3.08	96.83	10.11	175.33	2997.69	103.87	65.30	12.55
saturated	10.57	4.94	32.34	60.54	21.39	7.79	2.25	110.71	7.06	94.97	1631.78	104.84	59.87	10.58
unsaturated	27.87	9.87	2176344.34	115.31	63009461.52	32.51	5.29	60.04	18.18	388.25	6616.55	101.30	79.69	17.77
FAEEs	27.79	12.63	2340120.00	93.92	28665002.80	24.31	4.43	82.47	16.50	269.29	876.53	103.37	67.85	15.67
saturated	22.07	13.73	42.99	73.54	20.66	11.24	4.49	98.64	8.54	160.73	1561.20	104.89	58.16	9.04
unsaturated	34.85	11.29	5225736.43	119.05	64012618.81	40.43	4.36	62.54	26.32	403.14	32.25	101.48	79.79	23.85
overall	19.86	8.60	1231734.24	82.24	21419136.32	18.11	3.57	91.60	12.44	209.57	2224.81	103.69	66.23	13.69

Considering only GC models for the FAMEs class, it was observed the following accuracy order: CGC (3.08%) < OE (6.29%) < NRR (10.11%) < YPP (14.56%) < JR (15.31%) < Morris (75.55%) < Souders (96.83%) < SR (175.33%) < VCL (2997.69%) < Thomas (596376.10%) < HST (17265600.26%).

The Ceriani et al. (CGC) model^55^ presented the best performance of all models, presenting a %AARD lower than 4.0%, corresponding to the maximum uncertainty reported at *P*_0_. These results were expected since the CGC model was proposed for fatty compounds. The OE model presented the second-best performance, which could be related to the density data estimated by Yen and Woods^64^ model used as input. It is important to note that models that use experimental data as inputs could improve their accuracy. NRR also showed good results, which should be related to its good description of the interactions between –CH_3_– and –CH_2_– groups with electronegative atoms.

To CSP models, the crescent %AARD order for FAMEs is presented as follows: da Silva 2f (12.55%) < LS (65.30%) < PS (103.87%). Compared to GC models, da Silva 2f was as accurate as NRR. Such performance can be related to the chosen reference fluids, ME-C4:0 and ME-C24:0, which contain the experimental database’s smallest and largest carbon numbers.

Analyzing the %AARD profile varying the FAMEs’ carbon number, it was observed that by increasing the carbon number from ME-C4:0 until ME-C24:0, the %AARD behaved similarly to an interpolation profile. Therefore, minimum and maximum %AARD values were found to be around ME-C4:0 and ME-C24:0.

Comparing the models’ performances predicting η for FAEEs, it was observed that the results for FAAEs class were worse than for FAMEs. Besides, all models presented similar results for both classes. Models such as Thomas (5225736.43%), Morris (119.05%), HST (64012618.81%), Souders (82.47%), SR (269.29%), VCL (876.53%), PS (103.37%), and LS (67.85%) still presented large deviations.

Figure 1 illustrates the distribution of %ARD and %AARD values as a function of carbon chain length. As shown in Figure 1a, most of the %ARD values obtained with the CGC model (27%) fall within the [0.0–1.0%] range, following a clear linear decreasing trend. Thus, as the range of %ARD values increases, the frequency of predictions within each interval declines. Upon examination of the predictions by carbon chain length (Figure 1b), it becomes evident that the CGC model does not exhibit a distinct accuracy pattern, maintaining consistent performance across all chain lengths studied. Nevertheless, FAEEs in the C_4_–C_10_ range observed the most significant deviations.

**Figure 1:**
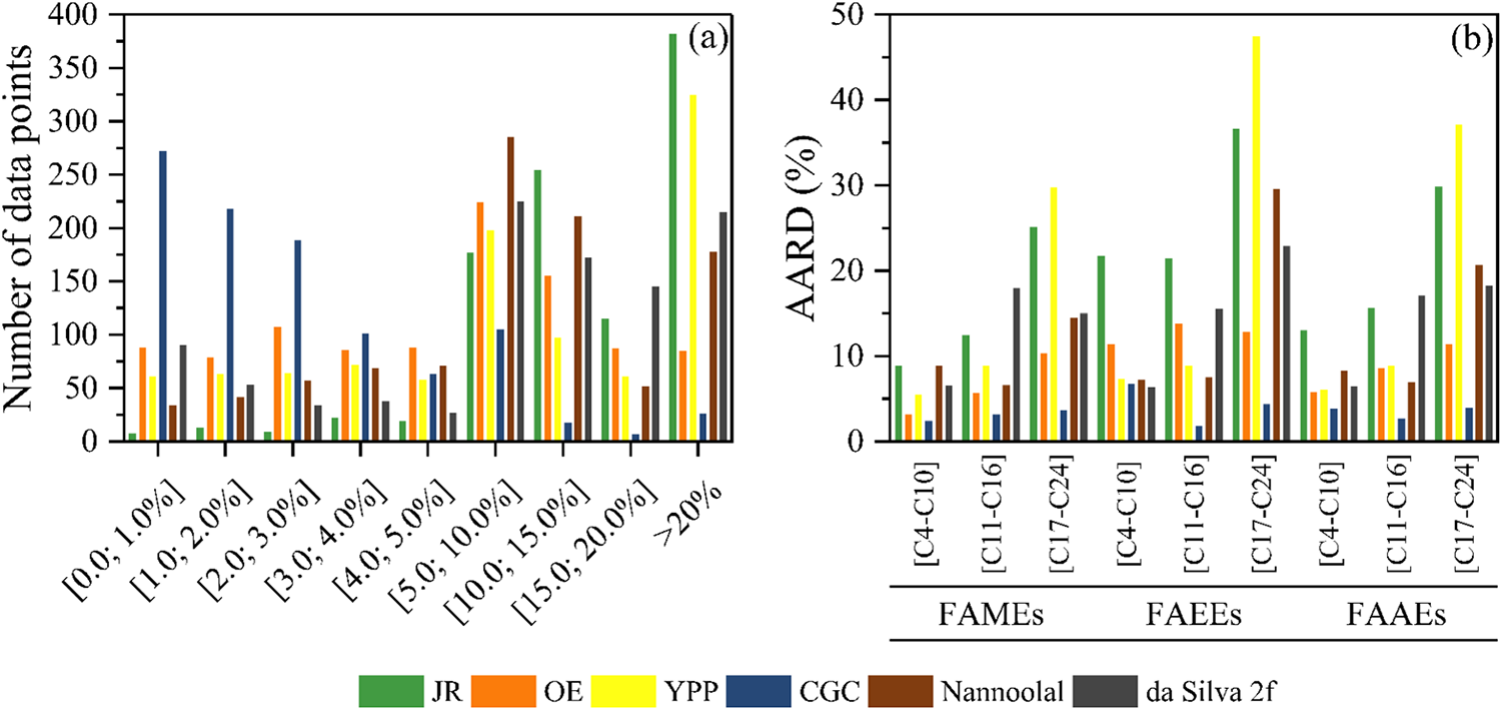
(a) ARD distribution and (b) %AARD profile obtained by the most accurate models predicting η considering the carbon number of FAAEs.

The JR, OE, YPP, and NRR models predominantly exhibited deviations in the [5.0%–10.0%] range, with relatively few predictions falling within the [0.0–1.0%] interval. Most predictions by da Silva 2f, OE, and YPP models within the lowest deviation interval [0.0–1.0%] corresponded mainly to shorter-chain FAAEs (C_4_–C_10_). Conversely, the NRR model achieved its highest accuracy for medium-chain FAAEs (C_11_–C_16_). Furthermore, all models, except NRR, consistently showed their smallest and largest prediction errors for short- (C_4_–C_10_) and long-chain FAAEs (C_17_–C_24_), respectively.

Figure 2 illustrates the influence of the temperature on the predictive performance of the models. Among the evaluated models, the CGC model provided the most reliable predictions across the temperature range investigated, exhibiting the lowest scattering of deviations. The OE model showed a similar deviation profile to CGC but with a slightly higher scatter. Both CGC and OE models presented their most significant outliers in the temperature interval between 263 and 301 K.

**Figure 2:**
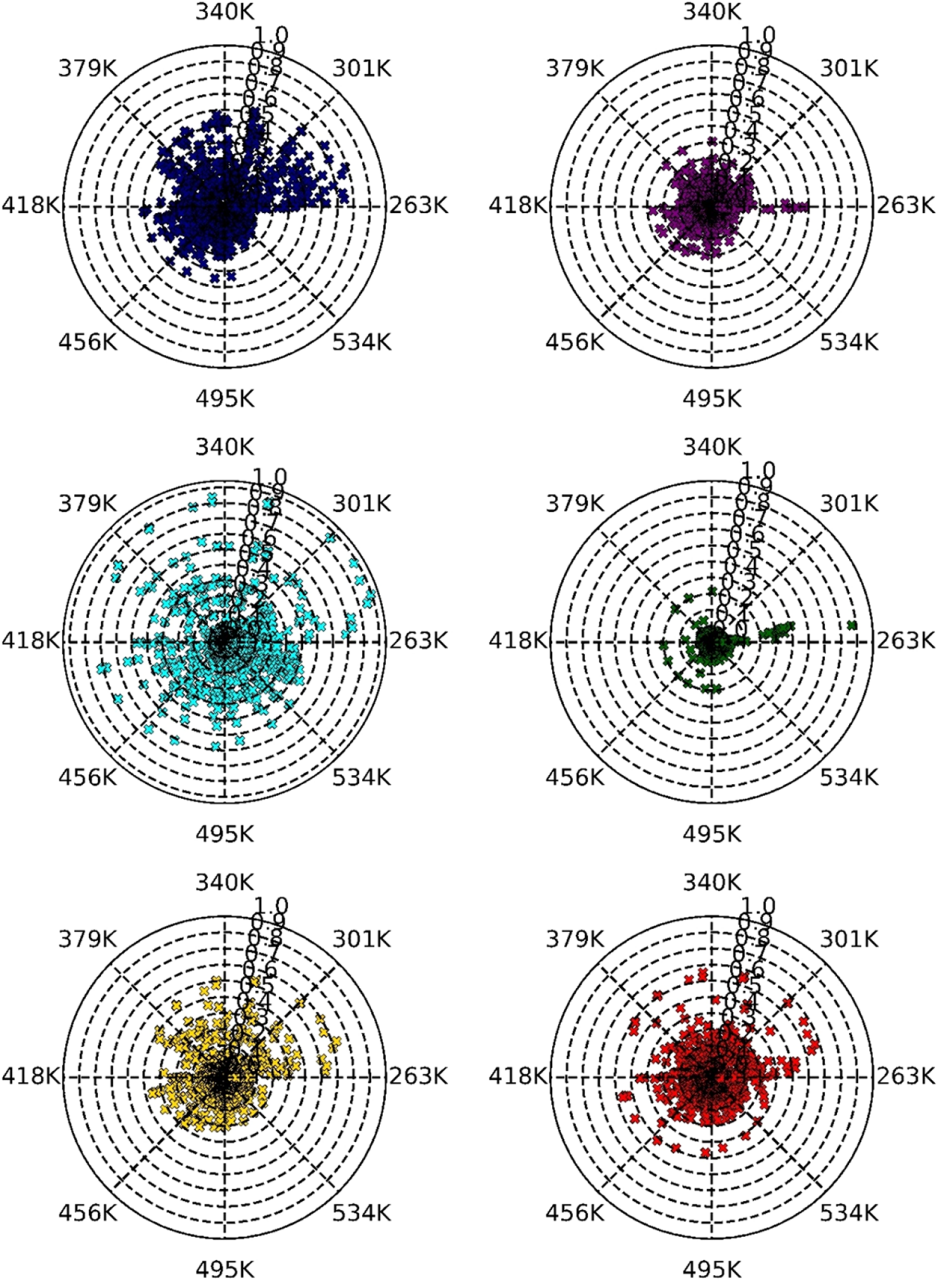
ARD distribution by the models in the temperature interval. The colors navy, purple, cyan, green, yellow, and red are the models JR, OE, YPP, CGC, NRR, and da Silva 2f, respectively.

The da Silva 2f and NRR models presented similar scattering patterns. Specifically, the NRR model exhibited more deviations between 263 and 379 K, with a reduction observed at higher temperatures (>379 K). In contrast, the da Silva 2f model presented a uniform deviation profile, lacking regions with particularly dense scattering, and thus maintained a consistent accuracy across all temperatures analyzed. The JR model showed a deviation behavior similar to that observed in the da Silva 2f and NRR models, but with greater overall scatter. Finally, the YPP model displayed the highest scattering among all of the evaluated models, highlighting its limited reliability in predicting η at the lowest and highest temperature extremes.

All models exhibiting an overall %AARD below 20% (i.e., JR, OE, YPP, CGC, NRR, and da Silva 2f) underwent the consistency tests described in Section 3.2. All successfully passed the homologous series test. However, during this evaluation, the CGC model unexpectedly exhibited discontinuities in the predicted viscosity profiles for some homologous pairs. This behavior is illustrated in Figure 3, where the viscosity profiles for selected saturated and unsaturated FAMEs predicted by the CGC and da Silva 2f models are compared.

**Figure 3:**
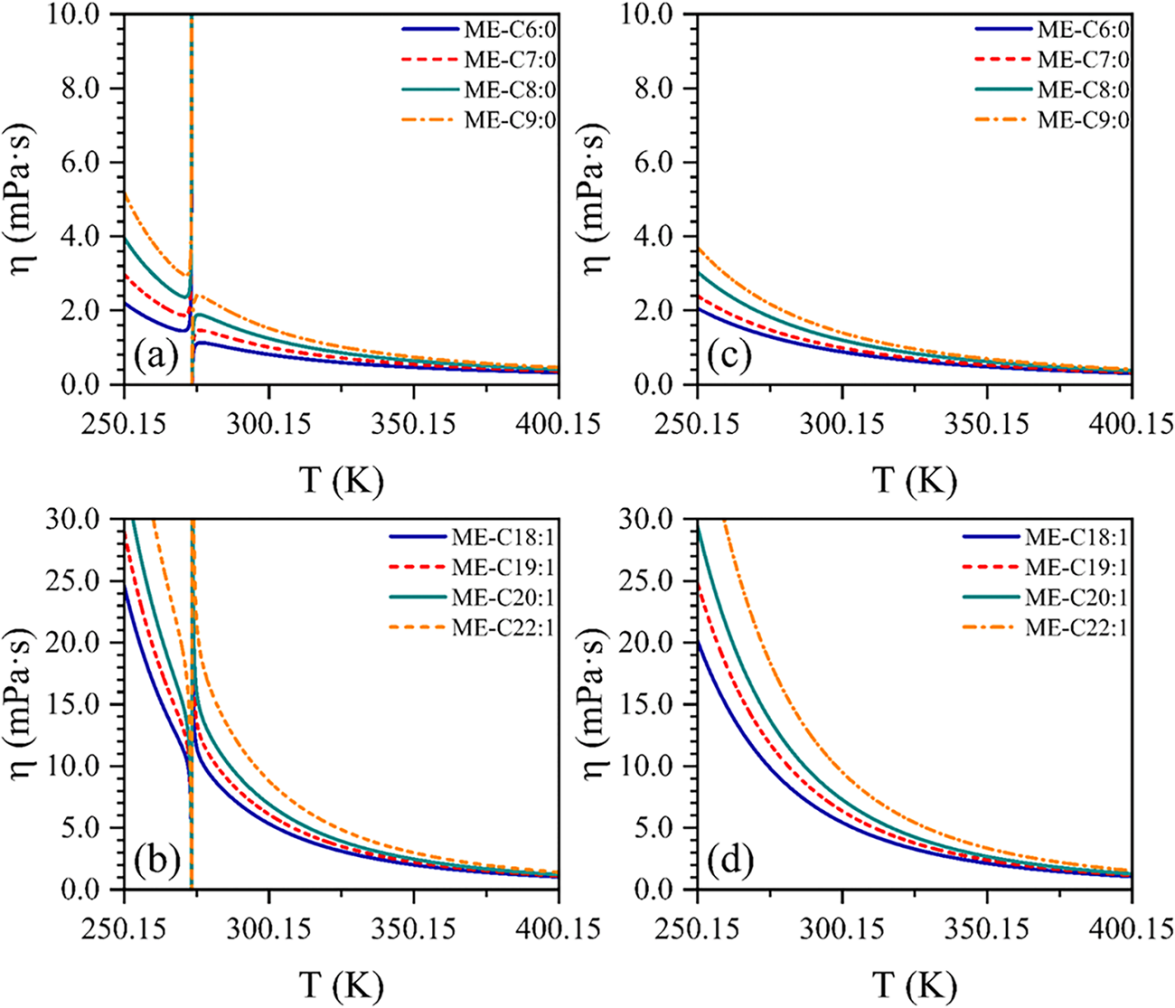
Comparison between generated profiles by CGC (a, b) and da Silva 2f (c, d) models for selected saturated and unsaturated FAMEs.

Figure 3a,c illustrates the anomalous behavior of the CGC model near 273.50 K, where the predicted viscosity values tend toward infinity. This behavior can be explained by a part of the model expression that exhibits a hyperbolic function. This issue could significantly impact the estimation of cold flow properties, such as cloud point, pour point, and cold filter plugging point, which typically occur near this temperature region.^72^ On the other hand, Figure 3b,d shows that the da Silva 2f model maintains continuous viscosity predictions across the entire temperature range evaluated, highlighting its reliability for estimating biodiesel viscosity in this extreme condition.

In addition to the homologous series test, the six best-performing models (JR, OE, YPP, CGC, NRR, and da Silva 2f) were evaluated using the “FAME/FAEE pair test”. This test employs eq 13 to confirm whether a FAEE consistently exhibits higher viscosity than its corresponding FAME (with an identical number of carbon atoms in the fatty chain) at the same temperature. A total of 36 model combinations (“packages”) were assessed, and only the following packages displayed consistent behavior: CGC/CGC (%AARD = 3.57%), OE/OE (%AARD = 8.60%), da Silva 2f/da Silva 2f (%AARD = 13.69%), YPP/YPP (%AARD = 18.11%), and JR/JR (%AARD = 19.86%). Additional information and complete results of all consistency tests are available in the Supporting Information.

### 4.2 Extension to High Pressures

Based on the %AARD analysis and the results of the consistency tests performed in previous sections, including the homologous series and ME/EE pair assessments, the proposed model (da Silva 2f) was selected as a reliable foundation for viscosity predictions under high-pressure conditions, as described by eqs 2 and ([Disp-formula eq3]). In this approach, the term η_0_ in eq 2 is calculated using the da Silva 2f model, while *D*_0_, *D*_1_, *D*_2_, and *C* are global constants derived from fitting experimental data.

Models validated against data sets distinct from those used for parameter fitting generally demonstrate enhanced reliability and improved predictive capabilities.^2,8,15^ Therefore, the high-pressure viscosity data presented in Table 1 were divided into two independent sets: a correlation data set and a prediction data set.

The complete high-pressure database consists of 408 experimental data points, randomly partitioned into the correlation and prediction data sets, each comprising 50% of the total data (204 points each). This random partition was performed using the *train_test_split* function from Python’s sklearn library.^73^ To estimate the global parameters, the *least_squares* function from Python’s SciPy library,^70^ which implements the Levenberg–Marquardt optimization algorithm, was employed. The objective function (*f*_obj_) used for parameter fitting was defined by eq 9. The optimization results for the global constants (*D*_0_, *D*_1_, *D*_2_, and *C*) of eq 2 are as follows: *D*_0_ = −1194.85, *D*_1_ = 7.62, *D*_2_ = −2.18 × 10^–2^, and *C* = 1.40.

Figure 4 presents the viscosity predictions generated by the high-pressure model for the correlation and prediction data sets and the corresponding relative deviations. Additionally, statistical analyses were conducted to thoroughly assess the accuracy and predictive reliability of the proposed model. It is important to highlight that the model constants (*D*_0_, *D*_1_, *D*_2_, and *C*) were optimized exclusively based on experimental data for selected FAMEs (C7:0-C10:0, C12:0, and C14:0) and FAEEs (C7:0-C14:0). As observed in Figure 4, the majority of relative deviations are within ± 10% and are randomly distributed around the baseline (0%), confirming the absence of systematic errors.

**Figure 4:**
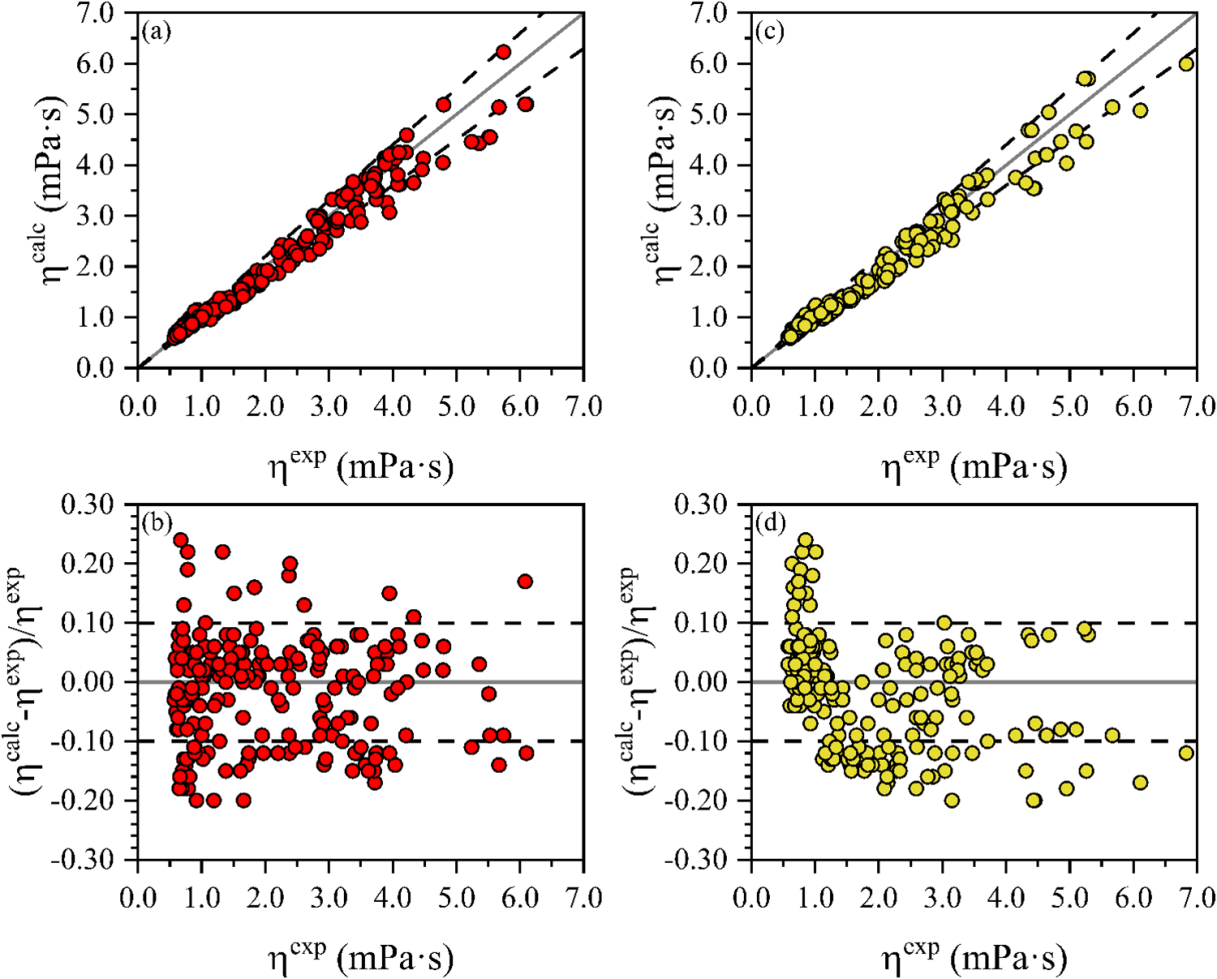
Plots of experimental versus calculated *P*-η-*T* for FAAEs with high-pressure data using the high-pressure model: (a) correlation set; (b) relative deviations for correlation set; (c) prediction set; (d) relative deviations for prediction set. Dashed lines correspond to ± 10% relative deviation.

Furthermore, the predictive performance of the high-pressure model was quantitatively evaluated using the root-mean-square error (RMSE), the most widely adopted parameter for predictive model analysis.^2^ The RMSE is calculated as follows

14 $$\mathrm{RMSE}=\sqrt{\frac{\sum_{i}^{N}\left(\eta_{i}^{\mathrm{calc}}-\eta_{i}^{\exp}\right)}{N}}$$

A lower RMSE value indicates a better predictive performance. In this study, the RMSE values obtained were 0.30 mPa·s for the correlation data set and 0.28 mPa·s for the prediction data set. The calculated %AARD values were 8.25 and 7.94% for the correlation and prediction data sets, respectively. These statistical indicators reinforce the robustness and reliability of the proposed high-pressure model. The complete set of predicted viscosity values used in this analysis is provided in the Supporting Information (SI).

## 5 Conclusions

In this work, a model based on the three-parameter Corresponding States Principle was proposed to estimate the dynamic viscosity of biodiesel-related esters (fatty acid methyl esters and fatty acid ethyl esters) over a wide range of temperatures and pressures. The proposed model exhibited strong accuracy, with average absolute relative deviations of 12.95% under atmospheric conditions and 7.94% under high-pressure conditions. A comprehensive analysis, including statistical indicators, deviation distribution, empirical trends, and temperature extrapolation, confirmed the model’s superiority, especially at low temperatures. Given its reliability, this model is highly recommended for future simulations, contributing to advancements in practical applications related to biodiesel.

## Supplementary Material



## Notes

Comprehensive information on the developed database; including all registered fatty acid alkyl esters; corresponding experimental data points with literature references; details of functional group assignments for each compound; refinement procedures at both low and high pressures; results of all consistency tests performed, and the outputs from all applied models, and including the calculated deviations for each compound (XLSX)

## Abbreviations

**Roman Letters**
AARD: average absolute relative deviation
RMSE: root-mean-square error
FAAE: fatty acid alkyl esters
ME: methyl esters
EE: ethyl esters
GC: group contribution
CSP: corresponding state principle
**Greek Letters**
η: dynamic viscosity
**Superscripts**
calc: calculated
exp: experimental

## References

[1] International Energy Agency . World Energy Outlook; OECD: Paris, 2024; Vol. 2024.

[2] Krishnasamy A., Bukkarapu K. R. (2021). A Comprehensive
Review of Biodiesel Property Prediction
Models for Combustion Modeling Studies. Fuel.

[3] Atabani A. E. E., Silitonga A. S. S., Badruddin I. A., Mahlia T. M. I. M. I., Masjuki H. H. H., Mekhilef S. (2012). A Comprehensive
Review
on Biodiesel as an Alternative Energy Resource and Its Characteristics. Renewable Sustainable Energy Rev..

[4] Hao S., Han X., Liu H., Jia M. (2021). Prediction and Sensitivity Analysis
of the Cetane Number of Different Biodiesel Fuels Using an Artificial
Neural Network. Energy Fuels.

[5] Demirbas A. (2009). Progress and
Recent Trends in Biodiesel Fuels. Energy Convers.
Manage..

[6] do
Carmo F. R., Evangelista N. S., Fernandes F. A. N., De Sant’Ana H. B. (2015). Evaluation of Optimal Methods for
Critical Properties and Acentric Factor of Biodiesel Compounds with
Their Application on Soave-Redlich-Kwong and Peng-Robinson Equations
of State. J. Chem. Eng. Data.

[7] Ramírez-Verduzco L. F. (2022). A Group
Contribution Method for Predicting the Alkyl Ester and Biodiesel Densities
at Various Temperatures. Sustainability.

[8] do
Carmo F. R., da Silva M. R. L., Alves A. A. A., Evangelista N. S. (2020). A New Method
for Predicting the Isobaric Heat Capacity of Biodiesel-Related Esters
Based on the Corresponding States Principle. Fluid Phase Equilib..

[9] Bird, R. B. ; Stewart, W. E. ; Lightfoot, E. N. Transport Phenomena, 2nd ed.; John Wiley & Sons, 2002.

[10] Yuan W., Hansen A. C., Zhang Q. (2009). Predicting
the Temperature Dependent
Viscosity of Biodiesel Fuels. Fuel.

[11] Verduzco L. F. R. (2013). Density
and Viscosity of Biodiesel as a Function of Temperature: Empirical
Models. Renewable Sustainable Energy Rev..

[12] Reid, R. C. ; Prausnitz, J. M. ; Poling, B. E. The Properties of Gases and Liquids, 4th ed.; McGraw-Hill, Inc: New York, 1987.

[13] Poling, B. E. ; Prausnitz, J. M. ; O’Connell, J. P. The Properties of Gases and Liquids, Fifith.; McGraw-Hill: New York, 2001.

[14] Cunico L. P., Ceriani R., Sarup B., O’Connell J. P., Gani R. (2014). Data, Analysis and Modeling of Physical Properties for Process Design
of Systems Involving Lipids. Fluid Phase Equilib..

[15] Evangelista N. S., Carmo F. R. d., de
Sant’Ana H.
B. (2017). Estimation of Vapor
Pressures and Enthalpies of Vaporization of Biodiesel-Related Fatty
Acid Alkyl Esters. Part 1. Evaluation of Group Contribution and Corresponding
States Methods. Ind. Eng. Chem. Res..

[16] Thorpe T. E., Rodger J. W. P. (1897). On the Relations between the Viscosity
(Internal Friction) of Liquids and Their Chemical Nature.—Part
II. Philos. Trans. R. Soc. London. Ser. A, Containing
Pap. Math. Phys. Charact..

[17] Batschinski A. J. (1913). Untersuchungen
Aber Die Innere Reibnng Der Flüssigkeiten. I. Z. Phys. Chem..

[18] Dunstan A. E., Thole F. B., Benson P. (1914). LXXX.—The
Relation between
Viscosity and Chemical Constitution. Part VIII. Some Homologous Series. J. Chem. Soc., Trans..

[19] Gill A. H., Dexter F. P. (1934). Viscosity of Esters
of Saturated Alphatic Acids: Relation
to the Synthesis of Fine Lubricating Oils. Ind.
Eng. Chem..

[20] Gouw T. H., Vlugter J. C. (1964). Physical Properties of Fatty Acid Methyl Esters. III
Dispersion. J. Am. Oil Chem. Soc..

[21] Trenzado J. L., Matos J. S., Segade L., Carballo E. (2001). Densities, Viscosities,
and Related Properties of Some (Methyl Ester + Alkane) Binary Mixtures
in the Temperature Range from 283.15 to 313.15 K. J. Chem. Eng. Data.

[22] Trenzado J. L., Matos J. S., González E., Romano E., Caro M. N. (2003). Study on
Properties Derived from Densities and Viscosities for the Ternary
Systems (Methyl Pentanoate or Methyl Heptanoate) + Octane + 1-Hexanol
and Their Binary Subsystems at Various Temperatures. J. Chem. Eng. Data.

[23] Gros A. T., Feuge R. O. (1952). Surface and Interfacial
Tensions, Viscosities, and
Other Physical Properties of Some n-Aliphatic Acids and Their Methyl
and Ethyl Esters. J. Am. Oil Chem. Soc..

[24] Matos J. S., Trenzado J. L., Santana S., Romaní L. (1996). Viscometric
Study of (an Aliphatic Methyl Ester + Heptane or Nonane) at the Temperature
298.15 K. J. Chem. Eng. Data.

[25] Bonhorst C. W., Althouse P. M., Triebold H. O. (1948). Esters
of Naturally Occurring Fatty
Acids - Physical Properties of Methyl, Propyl, and Isopropyl Esters
of C 6 to C 18 Saturated Fatty Acids. Ind. Eng.
Chem..

[26] Liew K. Y., Seng C. E., Oh L. L. (1992). Viscosities and Densities of the
Methyl Esters of Some N-Alkanoic Acids. J. Am.
Oil Chem. Soc..

[27] Knothe G., Steidley K. R. (2005). Kinematic Viscosity of Biodiesel Fuel Components and
Related Compounds. Influence of Compound Structure and Comparison
to Petrodiesel Fuel Components. Fuel.

[28] Wang X., Zhu S., Wang X. (2019). Liquid Viscosities for Methyl Hexanoate, Methyl Heptanoate,
Methyl Caprylate, and Methyl Nonanoate at High Pressures. J. Chem. Thermodyn..

[29] Habrioux M., Bazile J. P., Galliero G., Daridon J. L. (2016). Viscosities
of Fatty
Acid Methyl and Ethyl Esters under High Pressure: Methyl Myristate
and Ethyl Myristate. J. Chem. Eng. Data.

[30] Yao L., Hammond E., Wang T. (2008). Melting Points and Viscosities of
Fatty Acid Esters That Are Potential Targets for Engineered Oilseed. J. Am. Oil Chem. Soc..

[31] Pratas M. J., Pratas M. J., Freitas S., Oliveira M. B., Monteiro S. C., Lima A. S., Coutinho J. A. P. (2010). Densities
and Viscosities of Fatty
Acid Methyl and Ethyl Esters. J. Chem. Eng.
Data.

[32] Knothe G., Steidley K. R. (2007). Kinematic Viscosity
of Biodiesel Components (Fatty
Acid Alkyl Esters) and Related Compounds at Low Temperatures. Fuel.

[33] Pratas M. J., Freitas S., Oliveira M. B., Monteiro S. C., Lima A. S., Coutinho J. A. P. (2011). Densities and Viscosities of Minority
Fatty Acid Methyl
and Ethyl Esters Present in Biodiesel. J. Chem.
Eng. Data.

[34] Ceriani R., Gonçalves C. B., Rabelo J., Caruso M., Cunha A. C. C., Cavaleri F. W., Batista E. A. C., Meirelles A. J. A. (2007). Group
Contribution Model for Predicting Viscosity of Fatty Compounds. J. Chem. Eng. Data.

[35] Sastry N. V., Jain N. J., George A., Bahadur P. (1999). Viscosities, Speeds
of Sound and Excess Isentropic Compressibilities of Binary Mixtures
of Alkyl Alkanoate-Hydrocarbons at 308.15 and 318.15 K. Fluid Phase Equilib..

[36] Indraswati N., Wicaksana F. M., Hindarso H., Ismadji S. (2001). Density and Viscosity
for a Binary Mixture of Ethyl Valerate and Hexyl Acetate with 1-Pentanol
and 1-Hexanol at 293.15 K, 303.15 K, and 313.15 K. J. Chem. Eng. Data.

[37] Djojoputro H., Ismadji S. (2005). Density and Viscosity
Correlation for Several Common
Fragrance and Flavor Esters. J. Chem. Eng. Data.

[38] Djojoputro H., Ismadji S. (2005). Density and
Viscosity of Binary Mixtures of Ethyl-2-Methylbutyrate
and Ethyl Hexanoate with Methanol, Ethanol, and 1-Propanol at (293.15,
303.15, and 313.15) K. J. Chem. Eng. Data.

[39] Liu X., Lai T., Guo X., He M., Dong W., Shang T., Yang W. (2017). Densities and Viscosities
of Ethyl Heptanoate and Ethyl Octanoate
at Temperatures from 303 to 353 K and at Pressures up to 15 MPa. J. Chem. Eng. Data.

[40] Knothe G. (2008). “Designer”
Biodiesel: Optimizing Fatty Ester Composition to Improve Fuel Properties. Energy Fuels.

[41] Sheu Y. W., Tu C. H. (2005). Densities, Viscosities,
Refractive Indices, and Surface Tensions
for 12 Flavor Esters from T = 288.15 K to T = 358.15 K. J. Chem. Eng. Data.

[42] Sheu Y.-W., Tu C.-H. (2006). Densities and Viscosities
of Binary Mixtures of Isoamyl Acetate,
Ethyl Caproate, Ethyl Benzoate, Isoamyl Butyrate, Ethyl Phenylacetate,
and Ethyl Caprylate with Ethanol at T = (288.15, 298.15, 308.15, and
318.15) K. J. Chem. Eng. Data.

[43] Habrioux M., Bazile J. P., Galliero G., Daridon J. L. (2015). Viscosities of Fatty
Acid Methyl and Ethyl Esters under High Pressure: Methyl Caprate and
Ethyl Caprate. J. Chem. Eng. Data.

[44] Boelhouwer, J. W. M. ; Nederbragt, G. W. ; Verberg, G. Viscosity Data of Organic Liquids. 1951; Vol. 2.

[45] Hamazaki T., Kobayashi S., Urakaze M., Yano S., Fujita T. (1985). Viscosities
of Some Triglycerides and Ethylester of Fatty Acids Frequently Found
in Cell Membranes. Biorheology.

[46] Yamawaki H. (2020). Pressure Dependence
of Viscosity for Methyl Oleate and Methyl Linoleate up to 400 MPa. Int. J. Thermophys.

[47] de
Medeiros P. Y. G., da Silva M. R. L., Evangelista N. S., da Silva W. K., do Carmo F. R. (2022). Estimation of the Density of Ionic
Liquids over a Wide Temperature and Pressure Range: A Detailed Comparison
between the Group Contribution Models Available in the Literature. Ind. Eng. Chem. Res..

[48] Daridon J.-L. (2022). Predicting
Speed of Sound in Fatty Acid Alkyl Esters and Biodiesels at High Pressure. Ind. Eng. Chem. Res..

[49] Baylaucq A., Comuñas M. J.
P., Boned C., Allal A., Fernández J. (2002). High Pressure
Viscosity and Density Modeling of Two Polyethers and Two Dialkyl Carbonates. Fluid Phase Equilib..

[50] Gmehling, J. ; Kolbe, B. ; Kleiber, M. ; Rarey, J. Chemical Thermodynamics for Process Simulation; Wiley-VCH: Weinheim, 2012.

[51] Joback K. G., Reid R. C. (1987). Estimation of Pure-Component Properties
from Group-Contributions. Chem. Eng. Commun..

[52] Thomas L. H. (1946). 114. The
Dependence of the Viscosities of Liquids on Reduced Temperature, and
a Relation between Viscosity, Density, and Chemical Constitution. J. Chem. Soc. (Resumed).

[53] Hsu H., Sheu Y., Tu C. (2002). Viscosity estimation at low temperatures
(Tr<0.75) for organic liquids from group contributions. Chem. Eng. J..

[54] Yinghua L., Peisheng M., Ping L. (2002). Estimation of Liquid
Viscosity of
Pure Compounds at Different Temperatures by a Corresponding-States
Group-Contribution Method. Fluid Phase Equilib..

[55] Ceriani R., Gonçalves C. B., Coutinho J. A. P. (2011). Prediction of Viscosities of Fatty
Compounds and Biodiesel by Group Contribution. Energy Fuels.

[56] Souders M. (1938). Viscosity
and Chemical Constitution. J. Am. Chem. Soc..

[57] Nannoolal Y., Rarey J., Ramjugernath D. (2009). Estimation of Pure Component Properties.
Part 4: Estimation of the Saturated Liquid Viscosity of Non-Electrolyte
Organic Compounds via Group Contributions and Group Interactions. Fluid Phase Equilib..

[58] Sastri S. R. S., Rao K. K. (2000). A New Method for
Predicting Saturated Liquid Viscosity
at Temperatures above the Normal Boiling Point. Fluid Phase Equilib..

[59] van
Velzen D., Cardozo R. L., Langenkamp H. (1972). A Liquid Viscosity-Temperature-Chemical
Constitution Relation for Organic Compounds. Ind. Eng. Chem. Fundam..

[60] Przedziecki J. W., Sridhar T. (1985). Prediction of Liquid
Viscosities. AIChE J..

[61] Stiel L. I., Thodos G. (1961). The Viscosity of Nonpolar
Gases at Normal Pressures. AIChE J..

[62] Evangelista N. S., Carmo F. R. d., de
Sant’Ana H.
B. (2018). Estimation of Physical
Constants of Biodiesel-Related Fatty Acid Alkyl Esters: Normal Boiling
Point, Critical Temperature, Critical Pressure, and Acentric Factor. Ind. Eng. Chem. Res..

[63] Álvarez V. H., Valderrama J. O. (2004). Método Modificado Lydersen-Joback-Reid
Para
Estimar Propiedades Críticas de Biomoléculas. Alimentaria.

[64] Yen L. C., Woods S. S. (1966). A Generalized
Equation for Computer Calculation of
Liquid Densities. AIChE J..

[65] Pitzer K. S. (1955). The Volumetric
and Thermodynamic Properties of Fluids. I. Theoretical Basis and Virial
Coefficients. J. Am. Chem. Soc..

[66] Pitzer K. S., Lippmann D. Z., Curl R. F., Huggins C. M., Petersen D. E. (1955). The Volumetric and Thermodynamic Properties of Fluids.
II. Compressibility Factor, Vapor Pressure and Entropy of Vaporization. J. Am. Chem. Soc..

[67] Pitzer K. S., Curl R. F. (1957). The Volumetric and
Thermodynamic Properties of Fluids.
III. Empirical Equation for the Second Virial Coefficient. J. Am. Chem. Soc..

[68] Teja A. S., Rice P. (1981). Generalized Corresponding
States Method for the Viscosities of Liquid
Mixtures. Ind. Eng. Chem. Fundam..

[69] Teja A. S., Thurner P. A., Pasumartl B. (1985). Calculation
of Transport Properties
of Mixtures for Synfuels Process Design. Ind.
Eng. Chem. Process Des. Dev..

[70] Virtanen P., Gommers R., Oliphant T. E., Haberland M., Reddy T., Cournapeau D., Burovski E., Peterson P., Weckesser W., Bright J., van der Walt S. J., Brett M., Wilson J., Millman K. J., Mayorov N., Nelson A. R. J., Jones E., Kern R., Larson E., Carey C. J., Polat İ., Feng Y., Moore E. W., VanderPlas J., Laxalde D., Perktold J., Cimrman R., Henriksen I., Quintero E. A., Harris C. R., Archibald A. M., Ribeiro A. H., Pedregosa F., van Mulbregt P., Vijaykumar A., Bardelli A. P., Rothberg A., Hilboll A., Kloeckner A., Scopatz A., Lee A., Rokem A., Woods C. N., Fulton C., Masson C., Häggström C., Fitzgerald C., Nicholson D. A., Hagen D. R., Pasechnik D. V., Olivetti E., Martin E., Wieser E., Silva F., Lenders F., Wilhelm F., Young G., Price G. A., Ingold G. L., Allen G. E., Lee G. R., Audren H., Probst I., Dietrich J. P., Silterra J., Webber J. T., Slavič J., Nothman J., Buchner J., Kulick J., Schönberger J. L., de Miranda Cardoso J. V., Reimer J., Harrington J., Rodríguez J. L. C., Nunez-Iglesias J., Kuczynski J., Tritz K., Thoma M., Newville M., Kümmerer M., Bolingbroke M., Tartre M., Pak M., Smith N. J., Nowaczyk N., Shebanov N., Pavlyk O., Brodtkorb P. A., Lee P., McGibbon R. T., Feldbauer R., Lewis S., Tygier S., Sievert S., Vigna S., Peterson S., More S., Pudlik T., Oshima T., Pingel T. J., Robitaille T. P., Spura T., Jones T. R., Cera T., Leslie T., Zito T., Krauss T., Upadhyay U., Halchenko Y. O., Vázquez-Baeza Y. (2020). SciPy 1.0: Fundamental Algorithms for Scientific Computing
in Python. Nat. Methods.

[71] Paduszyński K., Domańska U. (2012). A New Group
Contribution Method For Prediction of Density
of Pure Ionic Liquids over a Wide Range of Temperature and Pressure. Ind. Eng. Chem. Res..

[72] Van
Gerpen J. H., He B. B. (2014). Biodiesel and Renewable Diesel Production
Methods. Adv. Biorefineries.

[73] Pedregosa F., Gael V., Gramfort A., Michel V., Thirion B. (2012). Scikit-Learn:
Machine Learning in Python. J. Mach. Learning
Res..

